# Inhibition of USP1 induces apoptosis via ID1/AKT pathway in B-cell acute lymphoblastic leukemia cells

**DOI:** 10.7150/ijms.47597

**Published:** 2021-01-01

**Authors:** Xingyi Kuang, Jie Xiong, Tingting Lu, Weili Wang, Zhaoyuan Zhang, Jishi Wang

**Affiliations:** 1 Department of Hematology, The Affiliated Hospital of Guizhou Medical University, Guiyang 550004, P.R. China.; 2 Guizhou Province Hematopoietic Stem Cell Transplantation Center, The Affiliated Hospital of Guizhou Medical University, Guiyang 550004, P.R. China.; 3 Key Laboratory of Hematological Disease Diagnostic & Treat Centre of Guizhou Province, Guiyang 550004, P.R. China.; 4 School of Basic Medical Sciences, Guizhou Medical University, Guiyang 550025, P.R. China.

**Keywords:** B-cell acute lymphoblastic leukemia, USP1, SJB3-019A, ID1, PI3K/AKT pathway, apoptosis

## Abstract

Deubiquitylating enzyme ubiquitin-specific protease 1 (USP1) has been reported to be aberrantly overexpressed in cancers, and it plays a critical role in regulating various cellular processes, such as cell proliferation, apoptosis, and cell differentiation. However, the role of USP1 in B-cell acute lymphoblastic leukemia (B-ALL) remains largely undefined. USP1 expression in 30 newly diagnosed B-ALL patients was detected by real-time PCR and western blot. We found that USP1 was generally upregulated in the bone marrow cells derived from B-ALL patients. Knockdown of USP1 by siRNA decreased B-ALL cell growth and induced apoptosis. Similarly, pharmacological inhibition of USP1 by SJB3-019A significantly repressed cell proliferation and triggered B-ALL cell apoptosis. Finally, we found that inhibition of USP1 downregulated the expression of ID1 and p-AKT, and upregulated ID1 expression could reverse the suppressive effects of USP1 inhibitor in B-ALL cells. Taken together, these results demonstrate that USP1 promote B-ALL progression at least partially via the ID1/AKT signaling pathway, and USP1 inhibitors might be promising therapeutic application for B-ALL.

## Introduction

B-cell acute lymphoblastic leukemia (B-ALL), the most common type of malignancy in children and young adults, is a genetically heterogeneous disease that derives from B cell progenitors 1. In spite of the great advances in the treatment of B-ALL, tumor relapse in B-ALL is one of the leading causes of cancer-related death in childhood 2. Adults with B-ALL experience even higher relapse rates, with long-term event-free survival less than 50% 3. Until now, the therapy of refractory/recurrent B-ALL remains particularly challenging. Therefore, it is necessary to explore novel therapeutic approaches to improve the outcome of B-ALL patients.

Ubiquitination is a very common posttranslational modification, which is reversible. Deubiquitinases (DUBs) are responsible for removing ubiquitin moieties from ubiquitinated substrate proteins, thus reducing their proteasomal degradation and maintaining the balance between ubiquitination and deubiquitination 4. In addition, the aberrant expression or function of DUBs generally leads to the pathogenesis and progression of a series of cancers 5-7. Ubiquitin-specific protease 1 (USP1) is one of the best-characterized members of DUBs, playing a critical role in regulating DNA repair processes and cell differentiation 8. More importantly, the aberrant expression of USP1 is closely associated with the tumorigenesis and progression of multiple cancers 9-11. Cumulative evidences have shown the abnormal overexpression of USP1 in malignant tumors 6,12,13. Notably, downregulation of USP1 could inhibit cell proliferation and promote apoptosis in a variety of solid tumors 9,11,12. In hematological malignances, downregulation of USP1 inhibited the proliferation of multiple myeloma (MM) and myeloid leukemia cells and induced cell apoptosis 13,14. However, to date, the role of USP1 in B-ALL remains unclarified.

In this study, we examined the expression of USP1 in bone marrow mononuclear cells (BM-MNCs) of 30 B-ALL patients and 18 healthy donors. Afterwards, siRNA and small molecular inhibitor were used to downregulate USP1 expression, aiming to further investigate the functional significance of USP1 in B-ALL-derived cell lines. Finally, we explored the mechanisms of the biological effects of USP1 on B-ALL cells.

## Materials and methods

### Reagents and antibodies

USP1 inhibitor SJB3-019A and proteasome inhibitor MG132 were purchased from MedChem Expression (New Jersey, USA). Antibodies against USP1, AKT, p-AKT were obtained from Cell Signaling Technology (MA, USA). Antibodies specific for β-actin were purchased from MDL biotech (Beijing, China). Monoclonal antibody against ID1 was bought from Santa Cruz (Heidelberg, Germany).

### Patient samples

Primary human B-ALL patient bone marrow samples were collected from The Affiliated Hospital of Guizhou Medical University. Age-matched healthy donors at Hematopoietic Stem Cell Transplantation Center of The Affiliated Hospital of Guizhou Medical University were also included. Mononuclear cells were separated from bone marrow by Ficoll gradient centrifugation. This study was approved by the institutional review board of The Affiliated Hospital of Guizhou Medical University and all participants offered informed consent according to the Declaration of Helsinki. Patients' characteristics are provided in **Table 1.**

### Cell culture

Human B-ALL cell lines CCRF-SB, Sup-B15 and KOPN-8 were cultured in complete medium containing 10% fetal bovine serum (Tianhang Biotechnology, Zhejiang, China) and antibiotics (Invitrogen, Carlsbad, USA). CCRF-SB, Sup-B15 were cryopreserved in Key Laboratory of Hematological Disease Diagnostic & Treat Centre of Guizhou Province. KOPN-8 cell line was obtained from Beijing Jingzhun Medical Technology Co., Ltd.

### Cell viability assay

The cell viability was assessed using the cell counting kit-8 (CCK-8) test. In brief, B-ALL cells were inoculated into 96-well plates with a density of 5×10^3^ cells/well. Then the cells were cultured overnight and treated with different approaches. Afterwards, 10 μL of CCK-8 solution (Dojindo, Kumamoto, Japan) was added to each well and incubated at 37˚C for 2 h, and absorbance values at 450 nm were measured via spectrophotometer (Molecular Devices, Sunnyvale, California, USA). The cell survival rate (SR) was calculated according to the following formula: SR (%) = (OD Treatment /OD Control) ×100%. The GraphPad Prism 8 software (GraphPad Software, San Diego, USA) was used to measure the IC50 value.

### Apoptosis assay

Apoptosis was determined by Annexin V-FITC and propidium iodide (PI) double staining according to the manufacturer's instructions (7Sea Biotech, Shanghai, China). In brief, B-ALL cells of each group were harvested and washed with PBS once. Cells were then resuspended with 300 µl of binding buffer, and incubated with 3.5 μL of Annexin V-FITC at room temperature for 15 min and then with 5 μL of PI at 4 °C for 5 min. Flow cytometry (BD Biosciences, San Jose, CA, USA) was performed to assess cell apoptosis. Each experiment was performed in triplicate.

### Cell cycle analysis

Cells were harvested, washed with ice-cold PBS, fixed in 70% ethanol at 4 °C for more than 2 hours. Afterwards, the fixed cells were washed with PBS, and incubated with RNase and PI (7Sea Biotech) for 30 min at 37 °C in dark. The cell cycle distribution was analyzed by flow cytometry (BD Biosciences).

### Quantitative reverse transcriptase-polymerase chain reaction (qRT-PCR)

According to the supplier's recommended protocol, total RNAs were isolated from cells using Trizol reagent (Invitrogen, Carlsbad, CA, USA) and reverse-transcribed by the Revertaid First Strand cDNA Synthesis Kit (Thermo Scientific, Waltham, Massachusetts, USA). The quantitative PCR reaction were performed with a SYBR Green PCR Master Mix (TianGen Biotech, Beijing, China) on the ABI 7500 real-time PCR detection system for 10 min at 95 °C, followed by 40 cycles at 95 °C for 15 s and at 60 °C for 1 min. The sequences of primers were as follows: USP1 F: 5′-TCATTCAATGGTTCTGGCTTA-3′, USP1 R: 5′-GGATTATTTGCG-GTTGTGATG-3′; ID1 F: 5′-AGGGGGCAAGAGGAATTACG-3′, ID1 R: 5′-TAGGTGTGCA-GAGAGGAGCG-3′; β-actin F: 5'-GACATCCGCAAAGACCTG-3', β-actin R: 5'-GGAAGG-TGGACAGCGAG-3'.

### Western blot analysis

Cells were lysed using RIPA buffer (Solarbio Science & Technology, Beijing, China) supplemented with protease and phosphatase inhibitors (Beyotime, Shanghai, China) for protein extraction. Total proteins were separated on SDS-PAGE gel and transferred to PVDF membranes. Membranes were blotted with primary antibodies for 2 hours at room temperature, membranes were then washed with TBST and incubated in secondary anti-rabbit-HRP (Beyotime) or anti-mouse-HRP antibodies (MDL biotech) for 45 min at room temperature. All protein bands were detected using the ECL reagent (7Sea Biotech) on the Tanon 4200 automatic chemiluminescence image analysis system (Tanon, Shanghai, China).

### Immunofluorescence Staining

After treatment, B-ALL cells were harvested and centrifuged, then fixed with 4% paraformaldehyde for 15 min and washed with PBS 3 times. Cell membrane permeabilization was performed with 0.1% Triton-X 100 (Beyotime) for 30 min. Afterwards, cells were incubated with fresh goat serum (5%) in following 1 h, cells were probed with specific primary antibody against ID1 at 4 °C overnight. After washing with PBS 3 times again, cells were incubated with the corresponding fluorescent-labeled secondary antibody (Beyotime). Finally, DAPI (Beyotime) was used to stain the nuclei. Fluorescence images were captured under a fluorescence microscope (Leica DM4000B, Wetzlar, Germany).

### Small interfering RNA (siRNA)-mediated gene silencing

B-ALL cells were seeded at a density of 1×10^5^ cells/well into a 6-well plate and cultured before transfection. After 12 hours, CCRF-SB and Sup-B15 cells were transfected with synthesized siRNA specifically targeting human USP1 (USP1-siRNA) (TransheepBio, Shanghai, China) or human ID1 (ID1-siRNA) (Santa Cruz) using Lipo6000 Transfection Reagent (Beyotime) according to the manufacturer's procedures. Meanwhile, a scrambled siRNA served as a negative control (NC-siRNA) (TransheepBio). After 48 hours of transfection, the RNA and protein were extracted and analyzed respectively.

### Construction of recombinant lentiviral vectors and transfection

Recombinant lentivirus-V5-D-TOPO-ID1-EGFP (LV-ID1) and control vector lentivirus-V5‐D-TOPO-EGFP (LV-control) were constructed. At 30-50% confluence, typically 24 h after plating, B-ALL cells were transfected with LV-ID1 and LV-control using 5 µg/ml polybrene (Genechem, Shanghai, China). After 5 days of infection, B-ALL cells expressing green fluorescent protein (GFP) protein was evaluated using fluorescence microscopy and flow cytometry, and the cells stably overexpressing ID1 were confirmed by real-time PCR and western blot.

### Statistical analysis

All statistical analyses were performed with SPSS software 20.0. Data were presented as mean ± standard deviation (SD). Student's t test was utilized to derive statistical significance when only two groups were compared. For experiments involving multiple comparisons, we performed one-way ANOVA with the Tukey's test to evaluate differences. *P* < 0.05 was considered statistically significant.

## Results

### USP1 expression in B-ALL patients

**Figure 1:**
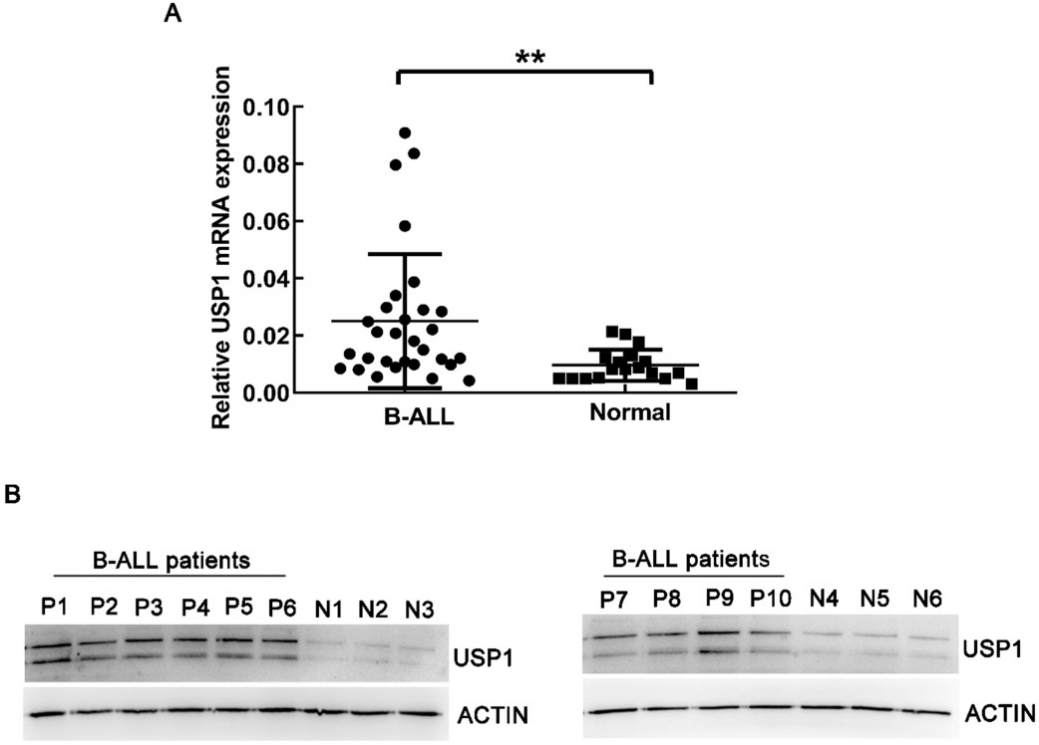
** Expression level of USP1 in B-ALL patients.** (**A**) Detection of mRNA expression level of USP1 in BM-MNCs from B-ALL patients and healthy donors using real-time PCR. **, *P* < 0.01 compared with healthy controls. (**B**) The protein levels of USP1 in B-ALL patients were determined by western blot. β-actin was used as an internal control.

Firstly, real-time PCR was utilized to detect the mRNA expression of USP1 in BM-MNCs from newly diagnosed B-ALL patients and healthy controls. As a result, the expression of USP1 was higher in B-ALL patients compared to that in healthy donors (Figure 1A, *P* < 0.01). Western blot analysis also revealed that the protein level of USP1 was higher in B-ALL patients in comparison to that in healthy controls (Figure 1B). These findings indicated a potential role of USP1 in the pathogenesis of B-ALL.

### The biological effects of USP1 inhibitor SJB3-019A on B-ALL cells

**Figure 2:**
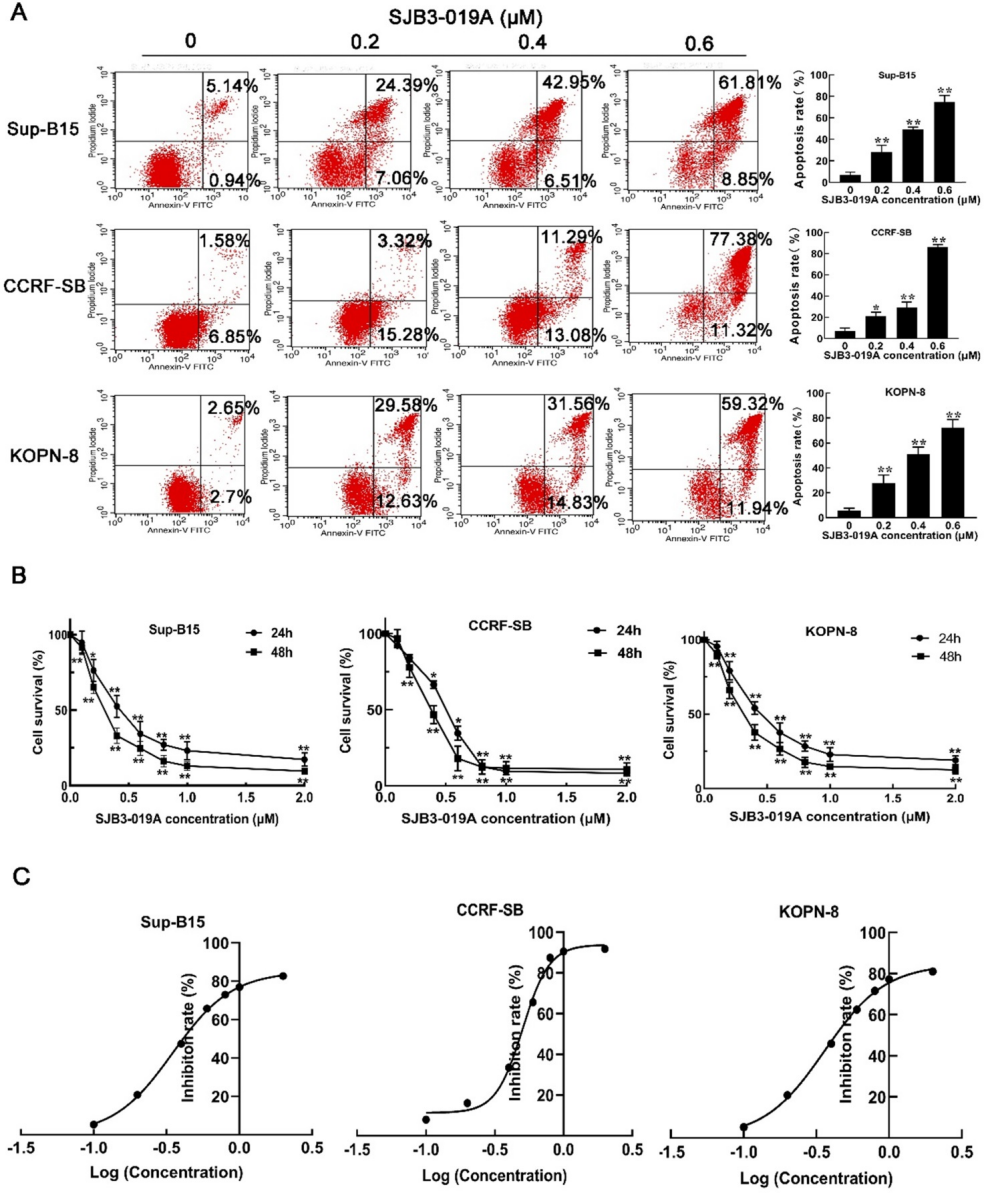
** SJB3-019A suppressed cell survival and induced apoptosis in B-ALL cells.** (**A**) Apoptotic rates of CCRF-SB, Sup-B15 and KOPN-8 were assessed by flow cytometry after treatment with 0, 0.2, 0.4, 0.6 µM SJB3-019A for 24 h. Data were presented as mean ± SD; *, *P* < 0.05 versus 0 µM, and **, *P* < 0.01 versus 0 µM group. (**B**) B-ALL cells were treated with various concentrations of SJB3-019A for 24 and 48 h, respectively, followed by cell viability evaluation by CCK-8 assay. Data were shown as mean ± SD; *, *P* < 0.05 versus 0 µM group; and **, *P* < 0.01 versus 0 µM group. (**C**) According to the OD_450_ values obtained from CCK-8 assay, the IC_50_ value was analyzed using Graphpad software 8.

To address the functions of USP1 on B-ALL cells, B-ALL cells were treated with different doses of SJB3-019A, a specific inhibitor of USP1. Consequently, CCK-8 assay showed that SJB3-019A suppressed the growth of B-ALL cells (CCRF-SB, Sup-B15 and KOPN-8) in a dose and time-dependent manner (Figure 2B), and these data demonstrated that Sup-B15 cells were the most sensitive to SJB3-019A (Sup-B15 IC50 = 0.349 μM, CCRF-SB IC50 = 0.504 μM and KOPN-8 IC50 = 0.360 μM) (Figure 2C). Moreover, SJB3-019A induced apoptosis of B-ALL cells in a dose-dependent manner (Figure 2A). To be specific, the apoptosis rate was 7.06% and 28.29% in the 0 μM and 0.2 μM SJB3-019A group, respectively, in Sup-B15 cells (*P* < 0.01). While in CCRF-SB cells, the apoptosis rate was 7.14% in the 0 μM SJB3-019A group, but increased to 20.88% in the 0.2 μM group (*P* < 0.01). In KOPN-8 cells, the apoptosis rate was 5.82% and 27.99% in the 0 μM and 0.2 μM SJB3-019A group, respectively (*P* < 0.01).

**Figure 3:**
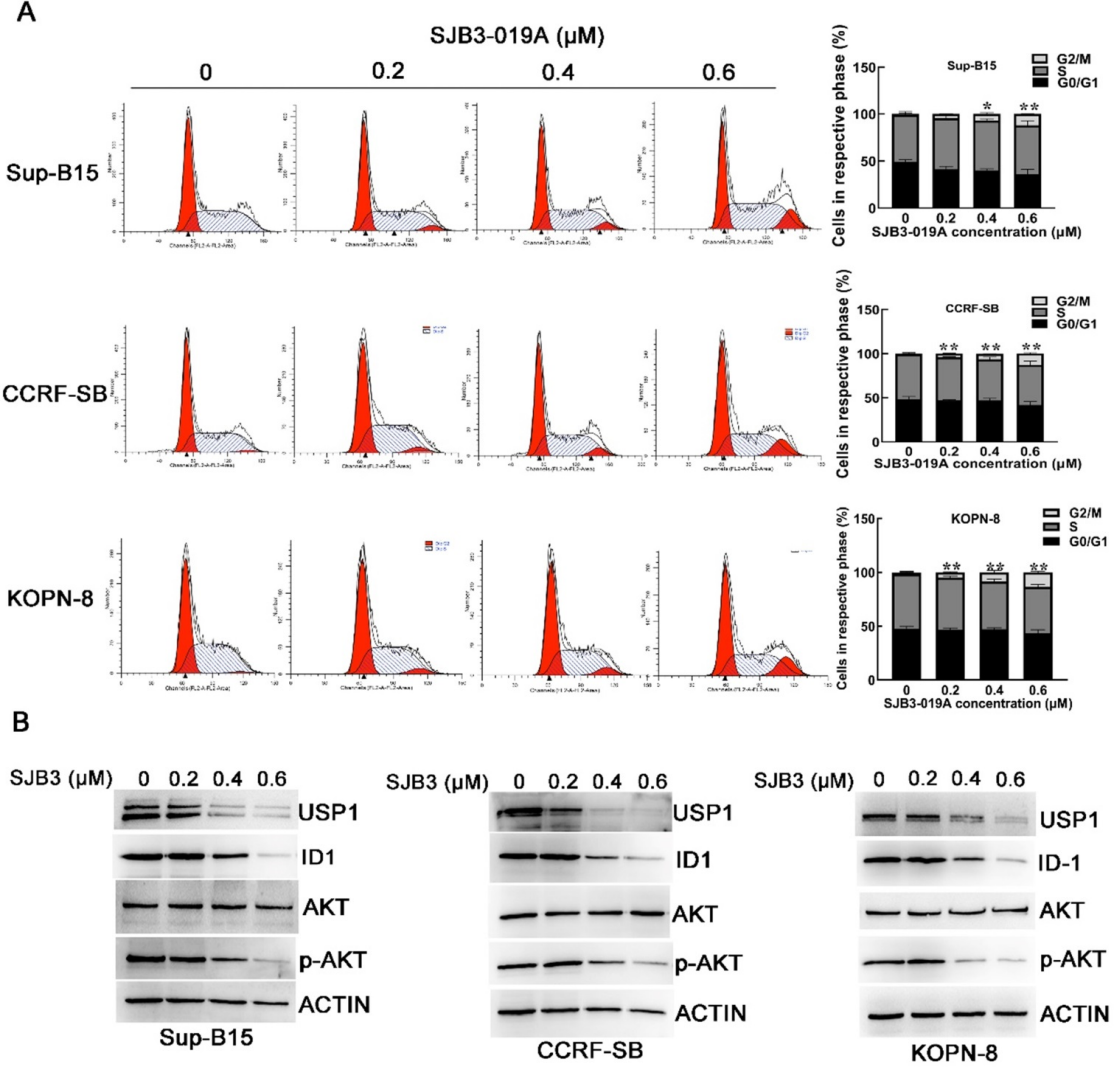
** Effects of SJB3-019A on cell cycle distribution and expression of USP1, ID1 and p-AKT.** (**A**) B-ALL cells were incubated with SMI-4a for 24 h, followed by flow cytometry to determine the cell cycle distribution. Data were presented as mean ± SD; *, *P* < 0.05 versus 0 µM group; and **, *P* < 0.01 versus 0 µM group. All experiments were performed in triplicate. (**B**) B-ALL cells were treated with SJB3-019A for 24 h, followed by detection of the protein expression of USP1, ID1, AKT and p-AKT using western blot analysis. β-actin was used as a loading control.

Previous studies have verified that SJB3-019A could cause G1/G0 cell cycle arrest in MM cells 12. Therefore, we examined the cell cycle distribution of B-ALL cells after treatment with SJB3-019A. However, cell cycle analysis implied that SJB3-019A induced G2/M phase arrest in B-ALL cells (Figure 3A). After treatment with 0.6 μM SJB3-019A, the percentage of cells in the G2/M phase increased from 0.90% to 12.17% in Sup-B15 cells (*P* < 0.01), and enhanced from 0.97% to 12.88% in CCRF-SB cells (*P* < 0.01).

### Silencing USP1 with siRNA inhibited cell growth

**Figure 4:**
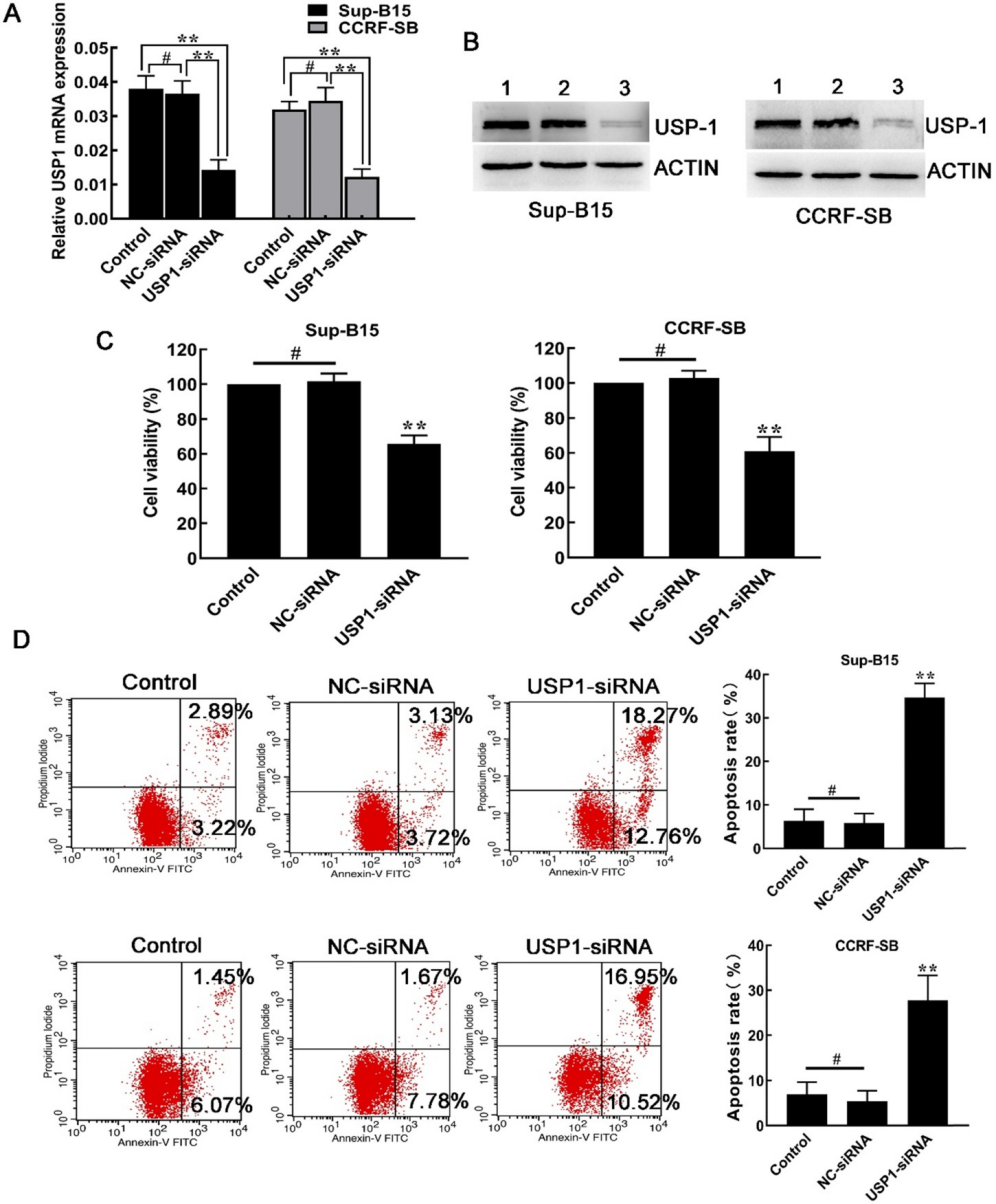
** Effects of USP1 siRNA in B-ALL cells.** (**A**) After transfection with USP1-siRNA, real-time PCR and was used to quantify the mRNA levels of USP1. **, *P* < 0.01; #, *P* > 0.05 versus the other two groups. (**B**) Western blot was used to detect the protein level of USP1. Lane 1, Control group; lane 2, NC-siRNA group; lane 3, USP1-siRNA group. (**C**) Depletion of USP1 inhibited the cell growth of CCRF-SB and Sup-B15 cells. Data were presented as mean ± SD; **, *P* < 0.01 versus other two group; ^#^, *P* > 0.05. (**D**) The effect of USP1 siRNA on apoptosis of CCRF-SB and Sup-B15 cells was determined by flow cytometry. Data were shown as mean ± SD; **, *P* < 0.01 in comparison to the control group; ^#^, *P* > 0.05.

Afterwards, siRNA was used to knock down the expression of USP1 in Sup-B15 and CCRF-SB cells. As a result, both mRNA and protein expression of USP1 were significantly inhibited in CCRF-SB and Sup-B15 cells (Figure 4A and 4B). Similar to the effects after SJB3-019A treatment, transfection of USP1-siRNA, but not NC-siRNA, decreased B-ALL cell viability (Figure 4C). In addition, knockdown of USP1 resulted in increased spontaneous apoptosis compared with other two control groups (Figure 4D). After transfection with USP1-siRNA, the percentage of apoptotic cells in Sup-B15 significantly increased from 5.86 ± 2.17% to 34.70 ± 3.22% (*P* < 0.01). In CCRF-SB cells, the percentage of apoptotic cells was 5.37 ± 2.38% in the NC-siRNA group, which increased to 27.76 ± 5.55% in the USP1-siRNA group (*P* < 0.01). Therefore, the above findings showed a critical role of USP1 in survival of B-ALL cells.

### Chemical or genetic inhibition of USP1 suppressed ID1/AKT pathway in B-ALL cells

**Figure 5:**
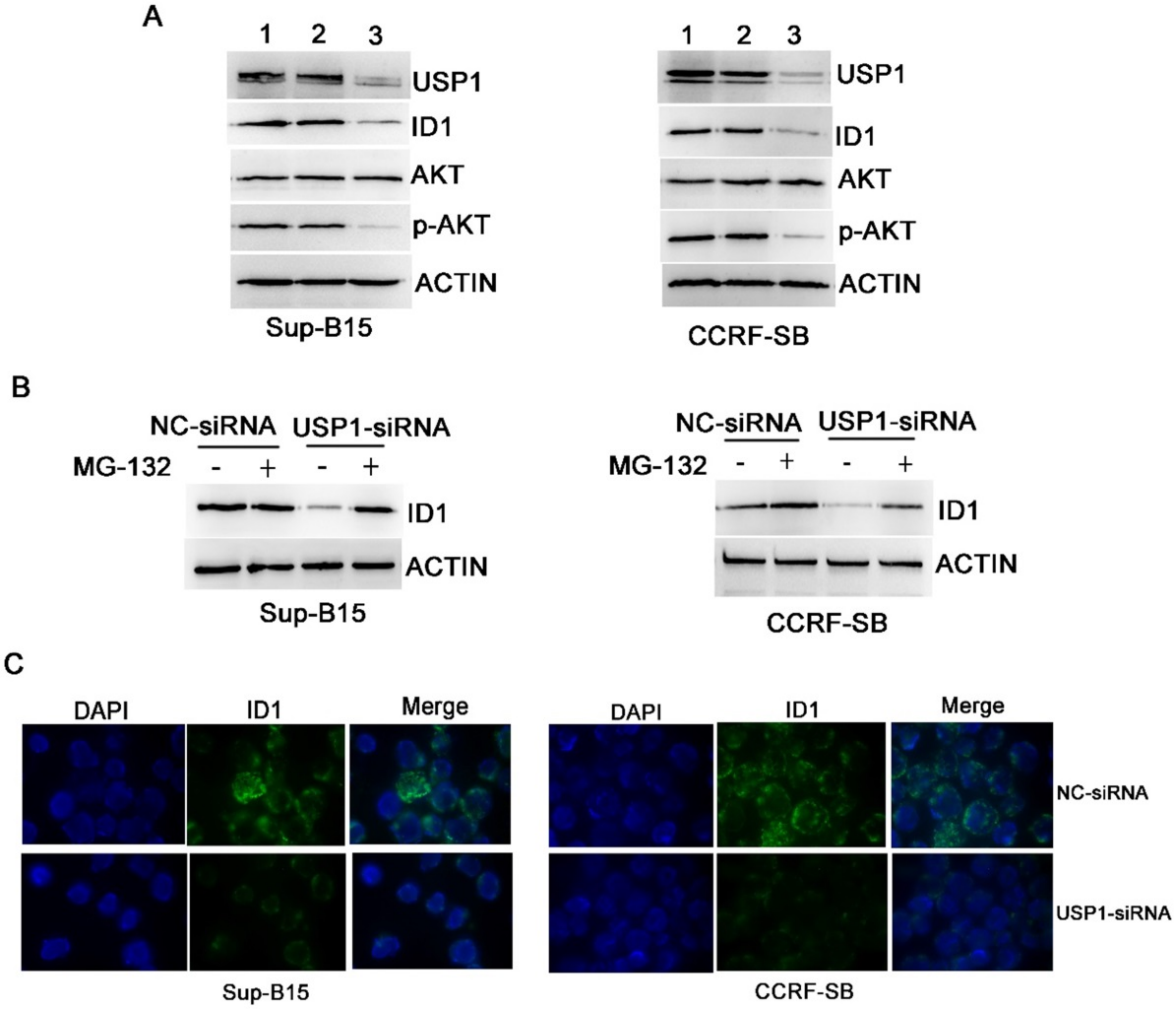
** USP1 regulated the expression of ID1/AKT pathway in B-ALL cells.** (**A**) After transfection with USP1-siRNA, the protein expression levels of USP1, ID1, AKT and p-AKT were detected by western blot. Lane 1, Control group; lane 2, NC-siRNA group; lane 3, USP1-siRNA group. (**B**) ID1 expression was analyzed by western blot in B-ALL cells with either NC-siRNA or USP1-siRNA in the presence or absence of MG-132. (**C**) Immunofluorescence staining of ID1 is performed as described in materials and methods. The images shown are under ×1000 magnification. The images are representative of three independent experiments.

Inhibitor of DNA binding 1 (ID1) protein is a known target of USP1 10, 13, and it was found significantly correlated with PI3K/AKT pathway 14. To further explore whether USP1 regulated the ID1/AKT axis in B-ALL cells, B-ALL cells were transfected with USP1-siRNA. As a result, USP1-siRNA transfection caused apparently decreased protein levels of USP1, ID1 and p-AKT in B-ALL cells, whereas the protein level of total AKT was not changed obviously (Figure 5A and 5C). Moreover, the administration of proteasome inhibitor MG-132 could rescue the protein level of ID1 in USP1-siRNA-treated B-ALL cells (Figure 5B), suggesting that USP1-siRNA decreased the protein expression of ID1 through proteasomal degradation. In addition, treatment with SJB3-019A in B-ALL cells attenuated the protein expression of USP1 in a dose-dependent pattern, and concomitantly reduced the protein levels of ID1 and p-AKT (Figure 3B).

### SJB3-019A induced apoptosis in B-ALL cells via ID1/AKT pathway

**Figure 6:**
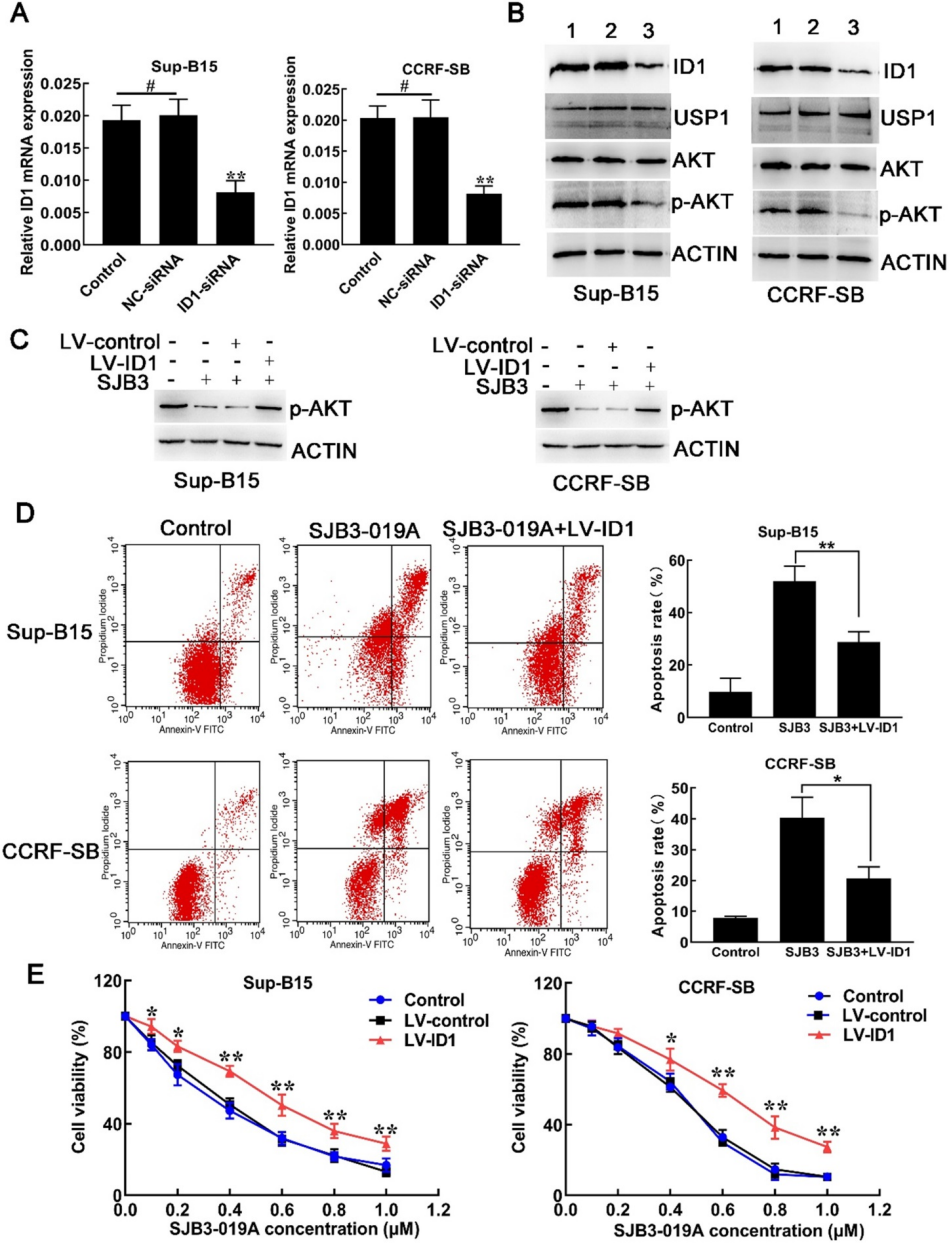
** SJB3-019A induced B-ALL cell apoptosis partially through ID1-mediated PI3K/AKT pathway.** (**A**) real-time PCR analysis of ID1 in B-ALL cells after transfection with either negative control siRNA or siRNA targeting ID1. **, *P* < 0.01 versus other two group; #, *P* > 0.05. (**B**) Western blot analysis of the protein expression of ID1, USP1, AKT and p-AKT in B-ALL cells. Lane 1, Control group; lane 2, NC-siRNA group; lane 3, ID1-siRNA group. (**C**) B-ALL cells transfected with either LV-control or LV-ID1 were incubated with SJB3-019A, followed by detection of the intracellular levels of p-AKT using western blot. (**D**) Apoptotic rates of CCRF-SB and Sup-B15 cells after ID1 regulation and SJB3-019A treatment were examined by flow cytometry. Data were presented as mean ± SD; *, *P* < 0.05, and **, *P* < 0.01. (**E**) B-ALL cells were infected with LV-control or LV-ID1, and incubated with different concentrations of SJB3-019A, followed by cell viability assessment by CCK-8 assay. *, *P* < 0.05, **, *P* < 0.01 versus the control group and LV-control group.

Similar to USP1, siRNA-mediated downregulation of ID1 also led to reduced level of p-AKT without effects on total AKT level. Downregulation of ID1 with siRNA caused decreased mRNA and protein expression of ID1, but did not decrease USP1 protein levels (Fig. 6A and 6B), indicating that USP1 functioned as the upstream of ID1 in B-ALL cells. To confirm the role of ID1 in suppressive effects of USP1 in B-ALL cells, lentivirus vector was used to upregulate the expression of ID1 in B-ALL cells. Flow cytometry showed that the percentage of GFP-positive cells was over 80% after transfection with lentivirus expressing ID1 (LV-ID1) (Supplementary Figure S1A), indicating successful infection. Next, the expression of ID1 was detected using real-time PCR and western blot. As a result, LV-ID1 infection significantly increased both mRNA and protein expression levels of ID1 (Supplementary Figure S1B and S1C). After infection with LV-ID1, the protein level of p-AKT was increased in SJB3-019A-treated cells (Figure 6C). More importantly, upregulated ID1 expression decreased apoptosis induced by SJB3-019A in B-ALL cells (Figure 6D). In addition, when B-ALL cells treated with SJB3-019A, upregulating ID1 significantly promoted cell viability (Figure 6E). Collectively, the present data demonstrated that SJB3-019A induced apoptosis at least partially through ID1-mediated AKT/PI3K pathway.

## Discussion

B-ALL, the most common type of ALL, is characterized by clonal expansion of developmentally arrested malignant B-cell precursors 2. Approximately 20% of B-ALL patients become resistant to chemotherapy during the therapeutic process 15. Moreover, patients with refractory/recurrent B-ALL have poor prognosis, without promising therapeutic effects in these patients 3. Therefore, it is necessary to develop novel therapeutic strategies targeting leukemia-specific molecular determinants for B-ALL.

The overexpression or hyper-activation of DUBs has been widely detected in various cancers, contributing to tumor development and progression 16. As a member of DUBs, USP1 is overexpressed in various types of cancers and is considered as an oncogene associated with cancer progression, metastasis and drug resistance 6, 11, 12. Recent studies have demonstrated that USP1 inhibition induced apoptosis and suppressed cell proliferation in acute myeloid leukemia (AML) and MM cells 12, 13. However, the exact roles of USP1 in B-ALL remain largely unknown. To this end, herein, we investigated the roles of USP1 in B-ALL cells. As a result, the USP1 expression was significantly higher in B-ALL patients than healthy controls. Because small molecule inhibitors are generally more suitable for clinical applications than gene knockdowns, we firstly determined the biological effects of USP1 using a small molecular inhibitor SJB3-019A. CCK-8 assay revealed that SJB3-019A inhibited cell vitality of B-ALL cells in a dose- and time- dependent manner, directly showing that USP1 may serve as a target for B-ALL therapy. Other two previous studies have confirmed the USP1 depletion caused cells arrest in G2/M phase 17, 18. In accordance with these findings, in our study, SJB3-019A treatment also caused G2/M cell cycle arrest in B-ALL cells. However, Deepika et al. 12 showed that SJB3-019A led to a G1/G0 cell cycle arrest in MM cells. These results indicate that SJB3-019A may affect different cell cycle stages depending on different types of cells. Similarly, siRNA-mediated downregulation of USP1 also resulted in increased B-ALL cell apoptosis and decreased cell growth, hence further implying a pro-survival role of USP1 in B-ALL.

ID1 protein is a member of the helix-loop-helix family of transcriptional regulatory proteins. As a known target of USP1 10, 13, the dysregulation of ID1 has been widely reported in multiple types of human tumors, which is essential for processes such as proliferation, cell migration and stem cell renewal 19-21. In B-ALL patients, high expression level of ID1 is associated with the poor outcome 22. Conditional deletion of ID1 prolongs the survival time of AML mice, while ID1 inhibitor obviously inhibits AML cell growth and promotes apoptosis, indicating that ID1 is a critical regulator in leukemogenesis 23. In our study, both USP1-siRNA and USP1 inhibitor (SJB3-019A) could decrease the protein level of ID1 in B-ALL cells. And the reduction of ID1 protein level caused by USP1-siRNA was mediated by proteasomal degradation, since the proteasome inhibitor, MG-132, rescued the expression of ID1 protein. More importantly, we found that upregulation of ID1 decreased the apoptosis caused by USP1 inhibitor SJB3-019A. These studies indicate that the suppressive role of USP1 in B-ALL cells is partially mediated by ID1.

The PI3K/AKT signaling pathway is involved in a wide range of physiological processes which is often dysregulated in tumorigenesis 24. In fact, the aberrant activation of PI3K/AKT pathway is very common in various types of cancers including hematological malignancies 25. Multiple studies have shown that the PI3K/AKT pathway is highly activated in B-ALL cells 26, 27, and inhibition of PI3K/AKT pathway leads to apoptotic activation in B-ALL cells 27. A previous study has proved that inhibition of ID1 significantly decreases p-AKT protein level in AML and osteosarcoma cells 20, 23. To further explore whether USP1 regulates the ID1/ AKT axis in B-ALL cells, the expression of USP1 and ID1 was knocked down in B-ALL cells, respectively. As a result, downregulation of USP1 and ID1 suppressed the PI3K/AKT pathway, as determined by the major reduction in p-AKT protein level. However, ID1 knockdown did not affect USP1, further suggesting USP1 is an upstream of ID1 in B-ALL cells. The present study indicated that USP1 silencing could downregulate the expression of ID1 and suppress the phosphorylation of AKT, suggesting that USP1 regulates PI3K/AKT signaling pathway in B-ALL progression, possibly by regulating ID1 expression. To further determine whether USP1-induced PI3K/AKT activation was mediated by the ID1 expression, B-ALL cells were treated with SJB3-019A and LV-ID1 or LV-control, respectively. Consequently, the upregulation of ID1 increased the protein level of p-AKT in SJB3-019A-treated B-ALL cells. Taken together, these findings demonstrate that inhibition of USP1 suppresses the activation of the PI3K/AKT pathway and promotes B-ALL cell apoptosis by downregulating the expression of ID1.

In conclusion, our current outcomes indicate the overexpression of USP1 in B-ALL patients. Genetic and pharmacologic inhibition of USP1 markedly suppress B-ALL cell growth and induce apoptosis. More importantly, inhibition of USP1 leads to the downregulated expression of ID1, further causing the inactivation of PI3K/AKT pathway. Therefore, USP1 inhibition presents as a novel therapeutic strategy for B-ALL, and future *in vivo* studies for USP1 inhibition are warranted.

## Supplementary Material

Supplementary figure S1.

## Figures and Tables

**Table 1:** Patients' characteristics

	Descriptive statistics
Total patients	30
Median age, years (minimum-maximum)	23 (1-48)
**Age**	
<10	6 (20%)
10-40	19 (63%)
>40	5 (17%)
**Gender**	
Female	11 (37%)
Male	19 (63%)
**White blood cells count (×10^9^/ L)**	
<10	15 (50%)
10-99	9 (30%)
≥100	6 (20%)
**Cytogenetics**	
Ph (+)	8 (27%)
TEL-AML1 (+)	1 (3%)
Normal	21 (70%)
**Immunophenotype**	
Pro-B-ALL	5 (17%)
Pre-B-ALL	7 (23%)
Common-B-ALL	18 (60%)

## Abbreviations

B-ALL: B-cell acute lymphoblastic leukemia
USP1: ubiquitin-specific protease 1
ID1: inhibitor of DNA binding 1
AML: acute myeloid leukemia
MM: multiple myeloma
BM-MNCs: bone marrow mononuclear cells
siRNA: small interfering RNA
CCK-8: cell counting kit-8
qRT-PCR: quantitative reverse transcriptase-polymerase chain reaction
