# Supplementary material for: Inhibition of USP1 induces apoptosis via ID1/AKT pathway in B-cell acute lymphoblastic leukemia cells
Source: Int J Med Sci. 2021 Jan 1;18(1):245–55. doi: 10.7150/ijms.47597 (PMC7738972; doi:10.7150/ijms.47597)
Supplement: Supplementary file 1 — Supplementary figure S1. [file ijmsv18p0245s1.pdf]

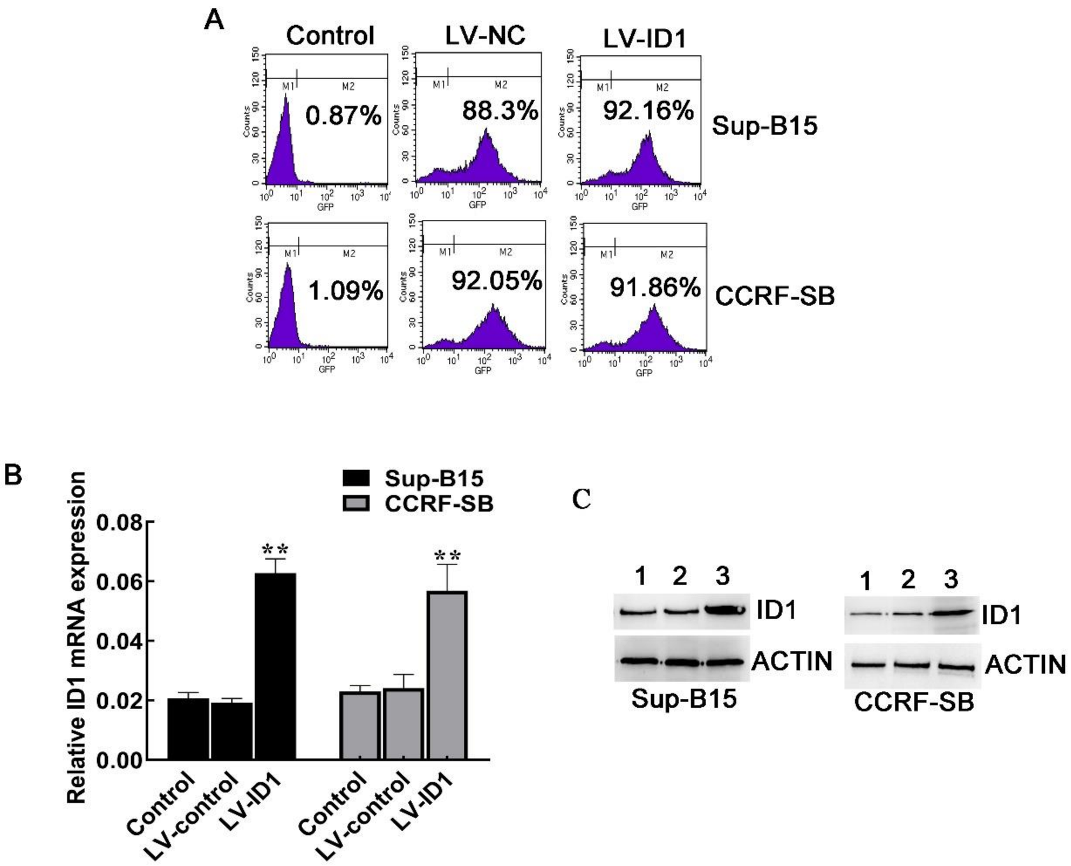

Figure S1. LV-ID1 successfully transfected B-ALL cells. (A) B-ALL cells were infected with LV-ID1, and the infection efficiency was examined by flow cytometry. (B) mRNA expression level of ID1 was significantly increased in LV-ID1-transfected group. **, $P < 0.01$ compared with other two groups. (C) Expression level of ID1 protein was assessed by western blot assay. Lane 1, Control group; lane 2, LV-control group; lane 3, LV-ID1 group.
